# Development of fatty acid metabolism-related models in lung adenocarcinomaA Review

**DOI:** 10.1097/MD.0000000000032542

**Published:** 2023-01-06

**Authors:** Wei Ye, Xingxing Li

**Affiliations:** a Department of Medical Respiratory, Wenzhou Municipal Hospital of Traditional Chinese Medicine, Wenzhou, Zhejiang Province, China; b Department of Oncology, Linping District First People’s Hospital, Hangzhou, Zhejiang Province, China.

**Keywords:** fatty acid metabolism, lung adenocarcinoma, prognosis

## Abstract

Lung adenocarcinoma (LUAD) is 1 of the common malignancy with a poor prognosis.

**Materials and Methods:**

Based on bioinformatics, the fatty acid metabolism model of LUAD was developed. We downloaded LUAD transcriptome data from the cancer genome atlas and gene expression omnibus databases. We used bioinformatics methods to construct a fatty acid metabolism-related predictive risk model to predict the prognosis of LUAD. We further explored the relationship between prognostic models and survival and immunity.

**Results:**

We identified 17 prognosis-related fatty acid-associated genes and constructed prognostic models. In the the cancer genome atlas cohort, the prognosis was worse in the high-risk score group compared to the low-risk score group. The ROC curve confirmed its accuracy. Subsequently, we used the gene expression omnibus database to confirm the above findings. There were differences in immune infiltrating cell abundance and immune function between the high-risk score group and low-risk score group. The immune dysfunction and exclusion (TIDE) based algorithm showed that the low-risk score group was more suitable for the immune treatment.

**Conclusion:**

Fatty acid metabolic patterns can deepen the understanding of the immune microenvironment of LUAD and be used to guide the formulation of immunotherapy protocols.

## 1. Introduction

Lung cancer is the main cause of cancer-related deaths worldwide with a poor prognosis (1). Non-small cell lung cancer (NSCLC) occupies a large category of lung cancer, and lung adenocarcinoma (LUAD) is the most common histological type of NSCLC, with the majority of LUAD patients diagnosed as being in an advanced stage.^[1]^ Although advances have been made in platinum based chemotherapy and targeted therapy with tyrosine kinase inhibitors, their use is limited by their limitations.^[2]^ In contrast, cancer immunotherapy has emerged as another highly effective novel therapy, especially the immune checkpoint blockade (ICB) which plays a transgenerational role in NSCLC. Only some patients can derive lasting benefits from it.^[3]^ Accurate determination of patients’ treatment efficacy is conducive to specify an effective, individualized regimen.

Tumor microenvironment is often characterized by hypoxic, highly oxidative, acidic and nutrient-poor conditions due to the need for rapid tumor cell proliferation.^[4]^ Cancer cells exhibit metabolic characteristics that are different from those of normal cells.^[4]^ Besides glucose metabolism, lipid metabolism is a distinctive features of malignancy.^[5]^ In recent years, fatty acid metabolism(FAM) has played an important role in many biological functions, such as cell membrane formation, energy storage and transmission of cancer signaling molecules, et cetera.^[6]^ Several novel inhibitors of fatty acid metabolism process are in clinical trials and are expected to be a new therapeutic tool.^[7]^ Fatty acid metabolism enhances melanoma immunotherapy by stimulating tumor-infiltrating T lymphocytes.^[8]^ The fatty acid metabolic pathway can play an important role in the value-added process of cancer cells by affecting tumor immunity and regulating the tumor microenvironment, and may become a new target for immunotherapy of cancer.^[9]^ Better fatty acid metabolic pathway of LUAD can contribute to understand the pathogenesis mechanism of LUAD and provide a new direction for new therapeutic approaches.

This study analyzed transcriptomic information in LUAD samples to comprehensively assess FAM pattern and construct a fatty acid metabolism related-prognostic risk model. The prognostic risk model could assess the efficacy of immunotherapy well and evaluate the relationship between the prognostic risk model and immune infiltrating cells. Our findings can provide a new perspective for exploring the mechanism and treatment of LUAD.

## 2. Materials and Methods

### 2.1. Acquisition and processing of data

We downloaded transcriptomic data and corresponding clinical information of LUAD from the the cancer genome atlas (TCGA), including 535 cancer samples and 59 normal samples. The data type was high-throughput sequencing (HTSeq) - reads per kilobase transcript per million mapping (FPKM). Clinical information of the 535 LUAD samples contained sex, Clinical Staging (TNM) stage and survival information. We downloaded the gene expression omnibus (GEO) dataset (GSE72094) and the corresponding platform annotation file from the GEO database. Three sets of genes related to fatty acid metabolism (Hallmark fatty acid metabolism genes, KEGG fatty acid metabolism pathway and response group fatty acid metabolism genes) were obtained from the Molecular Signature Database v7.2 (MSigDB), and fatty acid metabolism-related genes were retrieved after excluding overlapping genes for further analysis (Table S1, Supplemental Digital Content, http://links.lww.com/MD/I246).

### 2.2. Differential gene analysis

The “Limma” package is used to analyze the fatty acid metabolism DEGs between LUAD and normal samples. FDR < 0.05 was considered filtering conditions. Fatty acid metabolism-related genes were extracted from the TCGA and GEO cohorts, and we combined survival information. The DEGs in the TCGA cohort were handled using Univariate COX model regression analyses to identify prognosis-related genes. We used the “mafttools”package to analyze mutations and correlations of prognosis-associated fatty acid metabolism genes.

### 2.3. Construction and vertification of a prognostic risk scoring model

First,we adpoted the TCGA cohort as the train set and the GEO cohort as the test set. Then, we used the “glmnet” package to screen the prognosis-associated fatty acid metabolism genes in the TCGA cohort using least absolute shrinkage and selection operator (LASSO)-COX analysis to develop a prognostic risk scoring model in the LUAD sample and calculate the risk score of each sample.

coef: regression coefficients of genes included in prognostic risk score model; ExpGene: expression of genes included in the prognostic model.

We divided all samples into high-risk and low-risk score groups according to the median value of risk score. The differences in survival (OS) between the high- risk and low-risk scoring groups were compared by the Kaplan-Meier method. The ROC curves was plotted to evaluate the reliability and applicability of the prognostic risk model using the survivaROC package. Finally, We need to validate the reliability and applicability of the prognostic scoring model by calculating the risk values of each sample in the test set and grouping them using the above median cutoff point. The differences in OS between the high- risk and low-risk scoring groups of the test set were compared by the Kaplan–Meier method. The ROC curves was plotted to evaluate the reliability and applicability of the prognostic risk model of the test set using the survivaROC package.

### 2.4. Principal component analysis (PCA) of prognostic risk scoring model

We used principal component (PCA) analysis by the Limma package to explore distinguishing capability between of prognostic risk score model. First, PCA analysis was performed on the expression of differentially expressed fatty acid metabolism-related genes. Then, PCA analysis was performed on the expression of genes included in the prognostic risk model. Finally, visualization was performed using the ggplot2 package based on the results of the previous principal components.

### 2.5. Relationship between risk scores and clinical information

First, we combined the risk score and the corresponding clinical information, which included sex, age, and AJCC-TNM. To determine whether the risk score and clinical characteristics could be independent predictors, a Univariate COX regression model analysis of age, sex, AJCC-TNM stage and risk score was performed using the “Survival” package. We performed a Multivariate COX regression model analysis of multiple factors mentioned above to exclude the disturbance of confounding factors. ROC curves were plotted using the “SurvivalRoc” package to compare the predictive power of clinical information and risk scores. To understand the relationship between clinical information and high and low-risk groups, the samples were divided into subgroups based on clinical characteristics (sex, age, pathological stage) to compare the differences in risk scores. We used the Wilcoxon rank-sum test to compare the differences between the 2 groups, and the Kruskal-Wails test was used to compare the differences among more than 2 groups. *P* value < 0.05 was considered statistically significant.

### 2.6. Columnar line diagram of predicted overall survival

Based on the TCGA dataset, column line plots including age, pathological stage (Stage), AJCC-T, and risk score were developed using the rms package. We drew calibration curves to evaluate the accuracy of the column line plots. Multivariate COX regression analysis was used to determine whether the column line graph could be a prognostic indicator.

### 2.7. GSVA analysis

GSVA analysis is an unsupervised method of evaluating the relationship between the change of the pathway or function of a gene set. The “c2.cp.kegg.v7.1.symbols” from the molecular signature database were used as reference gene sets, and we used the “GSVA” package to perform KEGG analysis of genes within the predictive risk model. FDR < 0.05 indicates that the enriched pathways are statistically significant.

### 2.8. Relationship between risk score groups and immunity

CIBERSORT is a quantitative analysis of 22 human immune cell subtypes based on the linear support vector regression principle. The Wilcoxon rank-sum test is used to compare the differences in the abundance of immune infiltrating cells between high and low-risk score groups. Immune dysfunction and exclusion (TIDE) is a computational method that mimics the 2 main mechanisms of tumor immune evasion, allowing the determination of the efficacy of ICB therapy and the identification of new ICBs.^[10]^ The TIDE (http://tide.dfci.harvard.edu/) algorithm was used to predict the responsiveness of high and low-risk score groups to ICB-free. *P* value < 0.05 was considered statistically significant.

### 2.9. Protein-protein interactions network analysis

First, we compared the differential genes (DEGs) between the high-risk and low-risk score groups by the “Limma” package, and DEGs with adjusted *P* value < 0.05 were considered significant. Then, the DEGs were imported into the String database(https://cn.string-db.org/), and the DEGs were analyzed with a median confidence level > 0.90 as the threshold. Then, we took advantage of Cytoscape software (version 3.7.2) to display the processing results. We used the cytoHubba plugin to identify the hub genes in the DEGs. KEGG analysis and GO analysis of DEGs were performed using the cluster Profiler package. Finally, we divided all samples into high expression group and low expression group according to the median expression of hub genes. We used KM analysis to compare whether there was a difference of survival between the 2 groups. Wilcoxon rank-sum test was used to compare the relationship between immune infiltrating cells of prognosis-related genes. *P* value < 0.05 was considered statistically significant.

### 2.10. Statistical analysis

Wilcoxon rank-sum test was used to compare the difference between 2 and Kruskal-Wails test to compare the difference among more than 2 groups. Kaplan-Meier analysis was used to compare the difference in survival between high-risk and low-risk score groups. Univariate and multivariate COX regression analyses were used to determine the independent prognostic factors of LUAD ROC curves were plotted to assess the validity of the prognostic risk models and column line plots. We performed all statistics in R (version 4.0.3).

## 3. Results

### 3.1. Differential analysis of normal and cancer tissue

**Figure 1.**
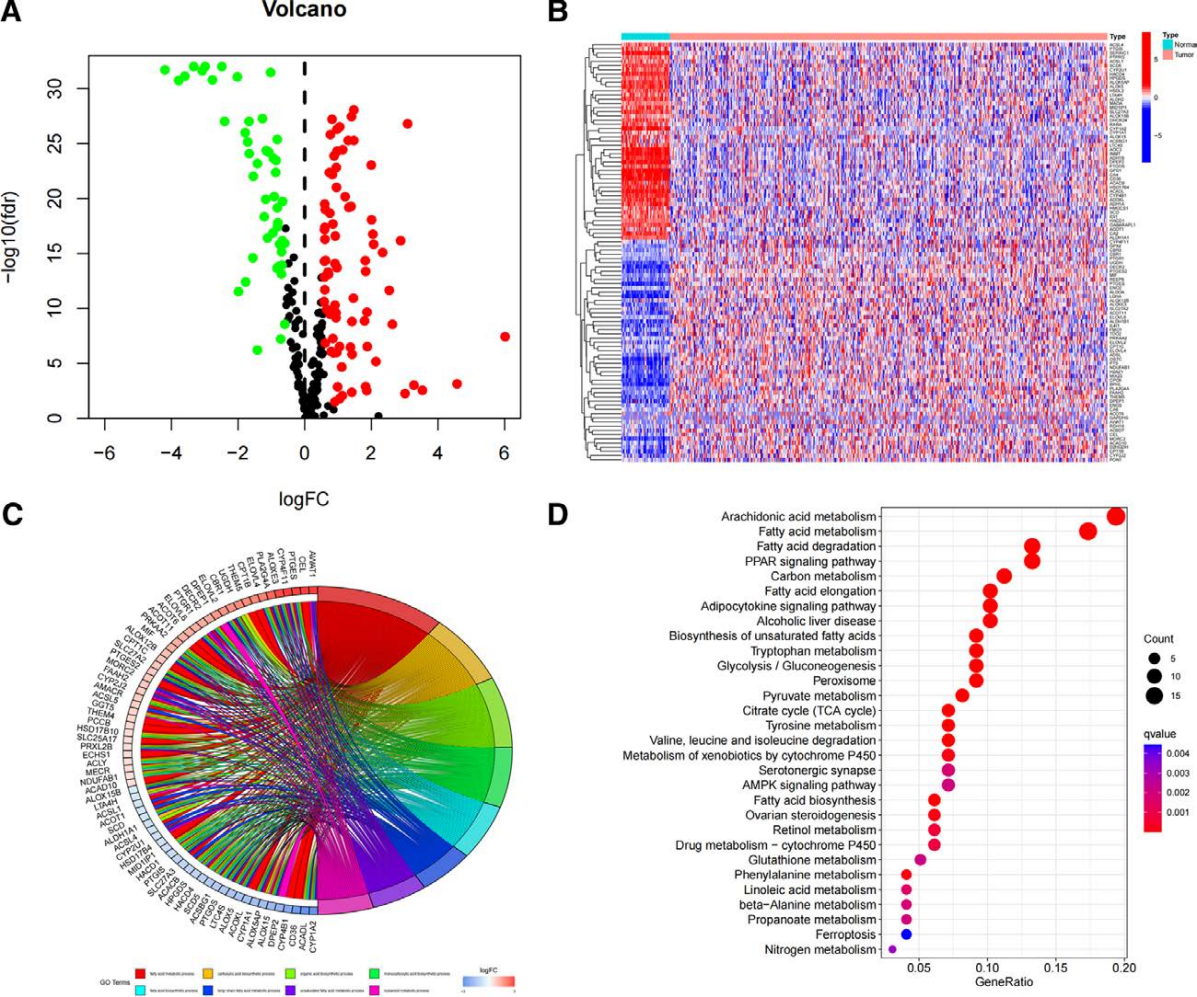
differentially expressed genes. (A) Volcano plot of differentially expressed genes: red dots indicate genes up-regulated in tumor samples, green indicates genes down-regulated in tumor samples, and black indicates non-responsive genes. (B) Heatmap of differentially expressed genes: light blue indicates normal samples, pink indicates red samples, red indicates genes upregulated in that sample, and blue indicates genes down-regulated. (C) The results of GO enrichment analysis. (D) The results of KEGG enrichment analysis.

We compared the expression quantity of fatty acid metabolism-related genes in cancer tissues and normal tissues, and a total of 126 genes was differentially expressed when screening based on the standard of FDR < 0.05 (Fig. 1A). Compared with normal samples, 47 genes were down-regulated, and 79 were up-regulated in tumor samples (Fig. 1B). The results of GO enrichment analysis showed that the enrichment was mainly in fatty acid metabolic process, organic acid metabolic process, long-chain fatty acid metabolic process, and unsaturated fatty acid metabolic processes (Fig. 1C). The results of KEGG enrichment analysis showed that the enrichment was mainly in fatty acid biosynthesis, degradation, and metabolism (Fig. 1D). The above indicates that fatty acid metabolic process plays an essential role in the pathology of LUAD.

### 3.2. Development of a predictive risk-score model

**Figure 2.**
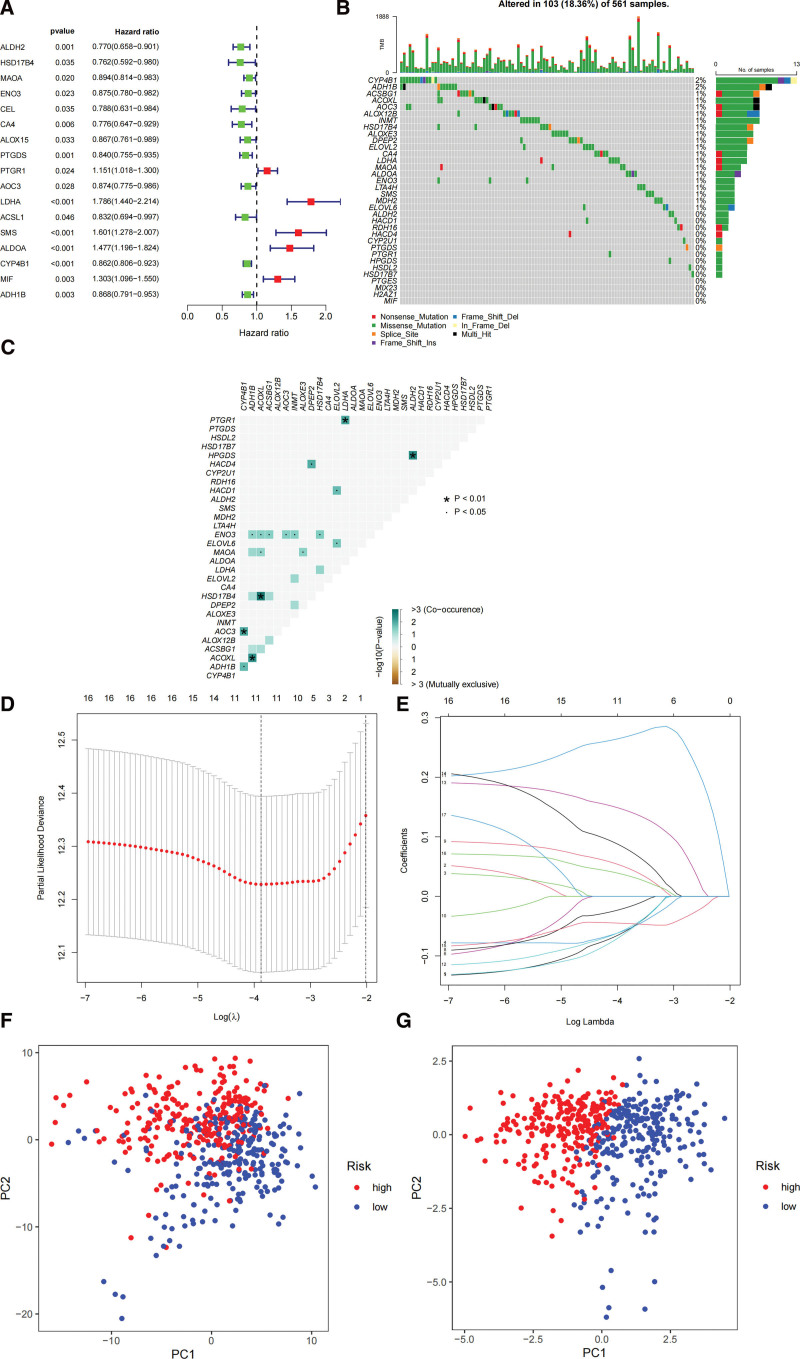
Development of prognostic risk model. (A) Forest plot of prognosis-related 17 fatty acid metabolism-related genes. (B) Mutation frequencies of 17 fatty acid metabolism genes in LUAD patients in the TCGA cohort. (C) Co-mutation analysis of 17 fatty acid metabolism-associated genes. (D) Identification of genes for prognostic risk models. (E) LAASO coefficients of fatty acid metabolism-associated genes. (F) Principal component analysis of fatty acid metabolism-associated genes in LUAD. (G) Principal component analysis of genes in the fatty acid prognostic risk model. LUAD = lung adenocarcinoma, TCGA = the cancer genome atlas.


We used a univariate COX regression model analysis performed on 126 DEGs of fatty acid (Fig. 2A). A total of 34 prognosis-associated genes was identified based on the standard of *P* value < 0.05. First, we summarized 34 prognostically relevant fatty acid-related genes (Fig. 2B). One hundred and 3 mutations were found in 561 LUAD samples (18.36%). We found the highest mutation frequencies in CYP4B1 and ADH1B. LDHA and PTGR1, ALDH2 and HPFGS, AOC3 and CYP4B1, HSD17B4 and ACOXL, AOC3 and ADH1B had mutational co-occurrence relationship (Fig. 2C). Genes were narrowed using LASSO-COX regression analysis (Fig. 2D and E). 17 genes were used to construct prognostic risk score models (Table S2, Supplemental Digital Content, http://links.lww.com/MD/I247). The risk score is calculated using the following formula:

$$\begin{array}{l} \begin{array}{ll} Riskscore= & (ALDH2*0.0232+ACSBG1*0.2143\\ & +\;ELOVL2*0.1754+ENO3*0.1259\\ & +\;CEL*0.0486+CYP2U1*0.2809\\ & +\;ACOXL*0.007+LDHA*0.2979\\ & +\;ACSL1*0.007+SMS*0.1141\\ & +\;ALOX12B*0.1534+ALDOA*0.0169\\ & +\;CYP4B1*0.0232+DPEP2*0.0733\\ & +\;ELOVL6*0.002+HPGDS*0.069\\ & +\;RDH16*0.1695) \end{array} \end{array}$$

The prognostic risk model was a good way to distinguish LUAD samples (Fig. 2F and G). The prognostic risk model was a good way to distinguish LUAD samples (Fig. 2F and G).

### 3.3. Relationship between risk scores and clinical characteristics

**Figure 3.**
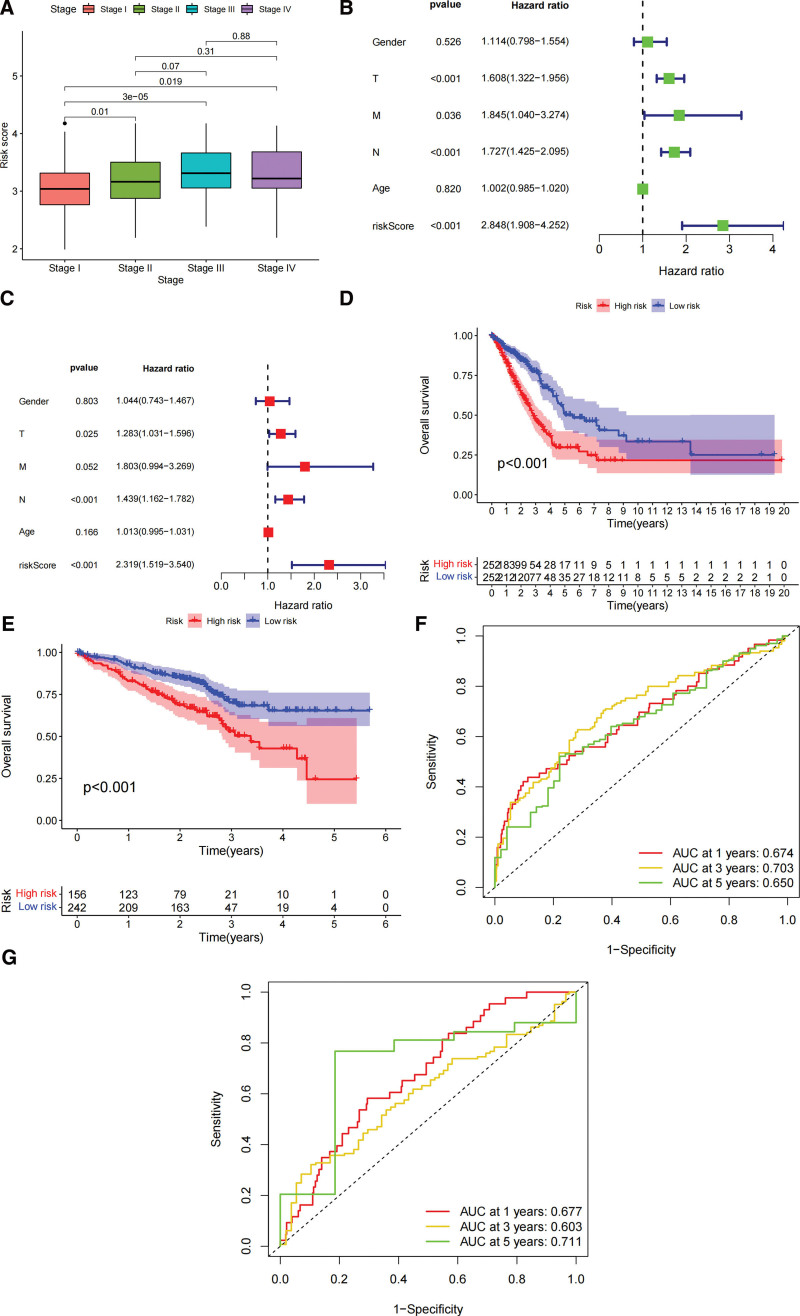
Predictive value of the fatty acid metabolism-related prognostic risk model for survival status of lung adenocarcinoma patients. (A) Relationship between risk score and pathological stage (Stage). (B) Analysis of Univariate COX regression model (clinical characteristics and risk score) in the TCGA cohort. (C) Multivariate COX regression model analysis (clinical features and risk score) in the GEO cohort. (D) Overall survival between high-risk and low-risk scores in the TCGA cohort. (E) Overall survival between high-risk and low-risk scores in the GEO cohort. (F) 1-year, 3-year, and 5-year predictive values of ROC curves for risk scores in the TCGA cohort. (G) 1-year, 3-year, and 5-year predictive values of ROC curves for risk scores in the GEO cohort. TCGA = the cancer genome atlas.

The samples in the TCGA cohort were divided into high-risk score groups and low-risk score groups according to the median value of risk scores. We analyzed the age, sex, and risk score distribution in the AJCC-TNM (Stage) of the TCGA cohort. There was no significant difference among risk scores, age and sex. Lower risk scores were associated with lower clinical staging (Stage) (*P* < .01, Fig. 3A). The factors related to survival in the Univariate COX model analysis were AJCC-T, AJCC-N and risk score (Fig. 3B). In the Multivariate COX model analysis, AJCC-T, AJCC-N and risk score could be independent prognostic factors for LUAD (Fig. 3C). K-M survival analysis suggested overall survival (OS) was longer in the low-risk score group than the high-risk score group (*P* < .001, Fig. 3D). The GEO cohort was categorized into high risk and low risk score groups according to the median score in the train sets (Table S3, Supplemental Digital Content, http://links.lww.com/MD/I248). There was a statistically significant difference in survival between the high and low risk score groups (*P* < .001, Fig. 3E). We also plotted the area under the ROC curve (AUC) at 1, 3, and 5 years to validate the accuracy of the prognostic risk model (Fig. 3F and G).

### 3.4. Construction of a column line graph for predicting survival

**Figure 4.**
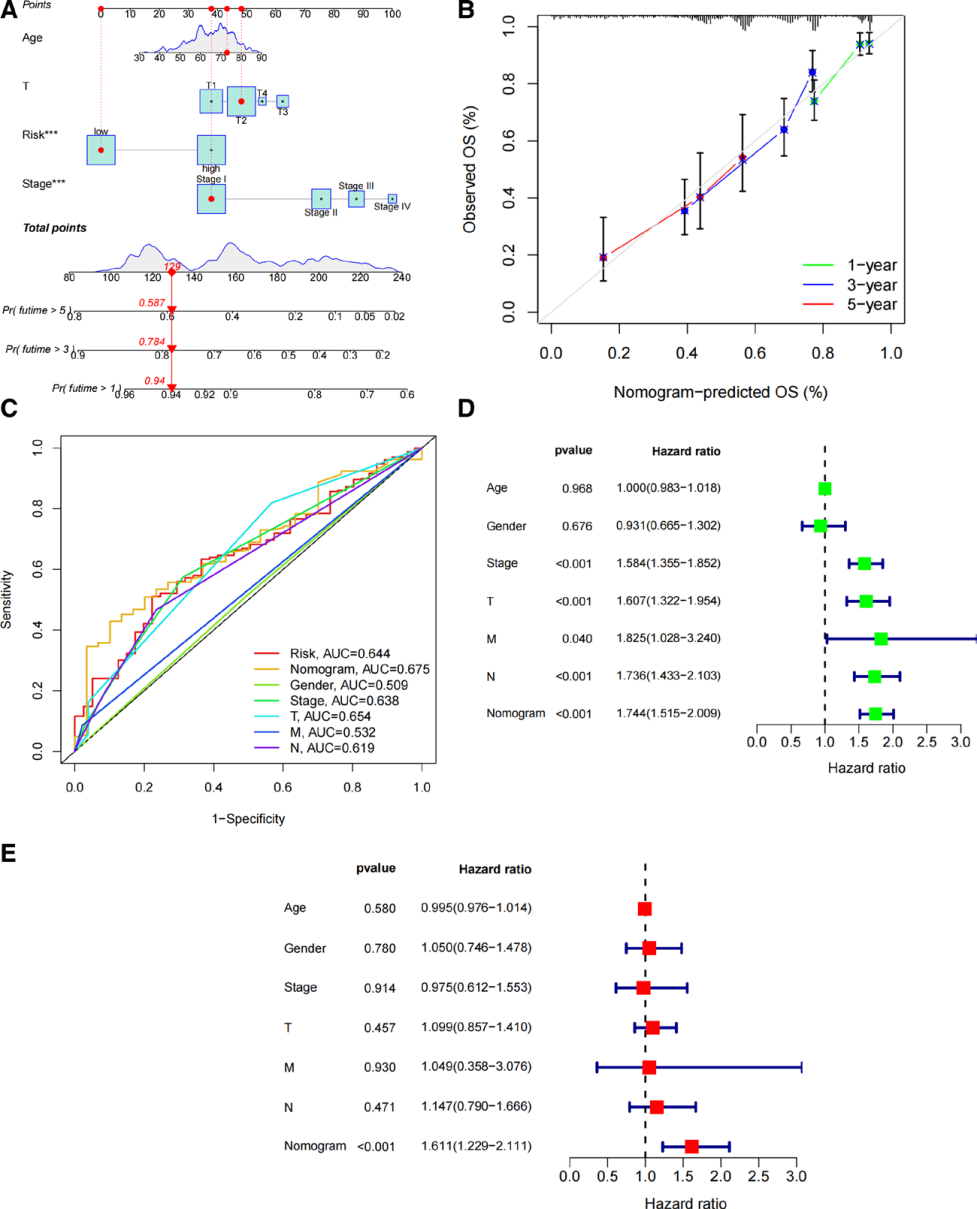
Predictive value of fatty acid metabolism-related risk scores combined with clinical characteristics in patients with TCGA-LUAD. (A) Column line graphs for predicting overall survival of patients in the TCGA cohort. (B) Calibration curves of the column line graphs. (C) AUC curves of column line graphs and clinical characteristics information. (D) One-way COX regression model analysis of column line graphs. (E) Multivariate COX regression model analysis of column line graphs. LUAD = lung adenocarcinoma, TCGA = the cancer genome atlas.

We also constructed a column line plot combining age, pathological stage (Stage), and risk score (Fig. 4A). 1-year, 3-year and 5-year calibration curves demonstrated that the column line plot could do well in predicting the predictive of LUAD (Fig. 4B). The AUC indicated that the column line plot (AUC = 0.675) had better prognostic value than single indicators such as sex (AUC = 0.509), T (AUC = 0.654), N (AUC = 0.619), M (AUC = 0.532), risk score (AUC = 0.644), and pathological stage (AUC = 0.638) (Fig. 4C). Multivariate COX regression model analysis suggests that this column line graph could be used as an independent prognostic factor (Fig. 4D and E).

### 3.5. Functional analysis

**Figure 5.**
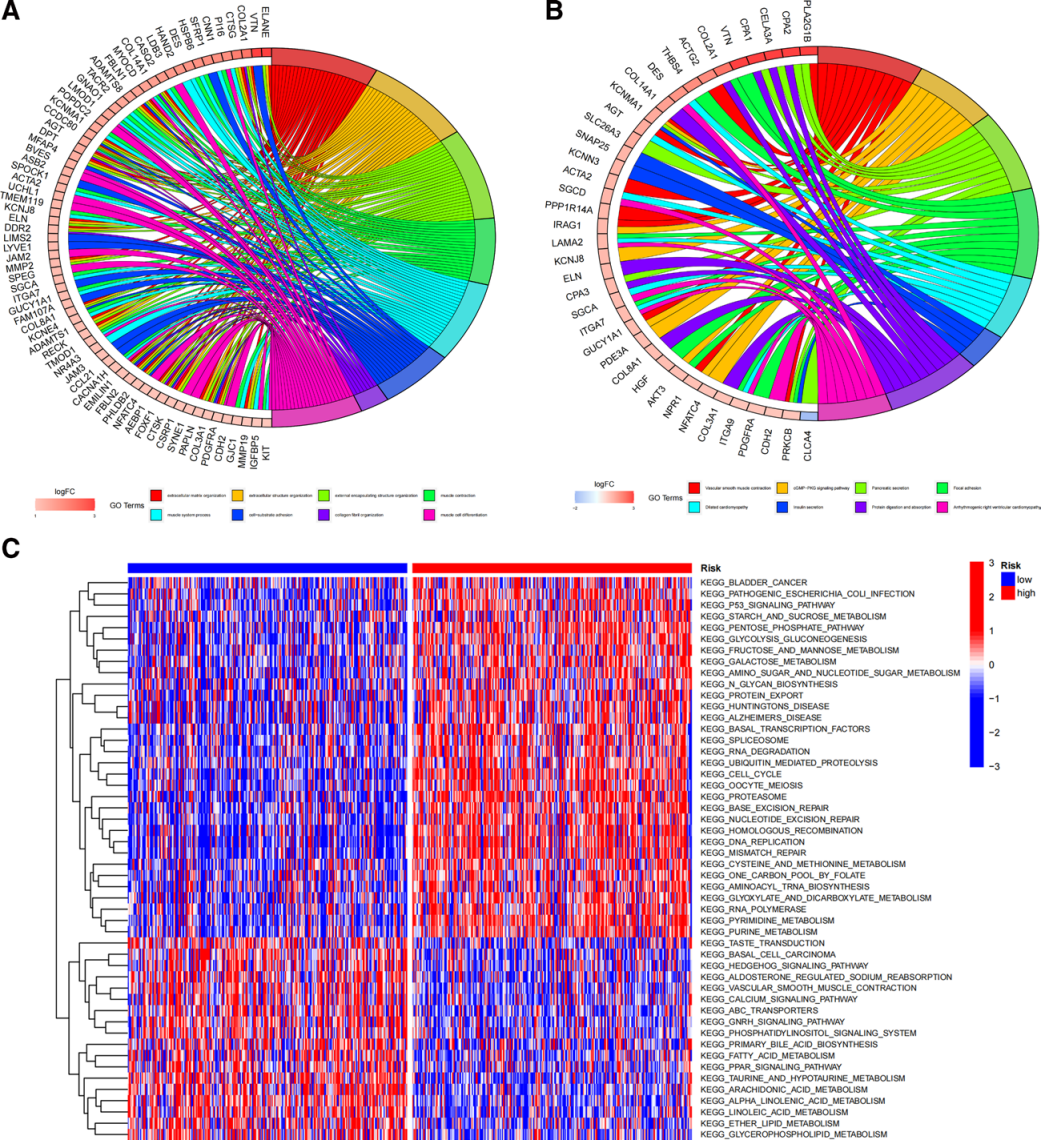
Functional analysis. (A) GO analysis of DEGs in high-risk and low-risk score groups. (B) KEGG analysis of DEGs in the high- risk and low-risk score groups. (C) GSVA analysis of DEGs in the high- risk and low-risk score groups.

KEGG and GO analyses of differential genes between high and low risk groups were performed using GOplot packages in order to better understand biological functions. The GO results showed that these genes were mainly involved in muscle contraction, extracellular matrix, cell-matrix adhesion, endopeptidase inhibitors, and integrin binding (Fig. 5A); the KEGG analyze showed that DEGs were mainly involved in the PI3K-Akt signaling pathway, the cGMP-PKG signaling pathway, WNT signaling pathway, cholesterol metabolism, fat digestion and absorption (Fig. 5B). The “c2.cp.kegg.v7.4.symbols” package was downloaded using the molecular signature database, and GSVA analysis was performed based on the gene set to explore the biological behavior of the 2 subgroups. The low-risk score group had more fatty acid metabolic pathways, such as fatty acid metabolism, lipid metabolism, linolenic acid metabolism, linoleic acid metabolism, and glycerophospholipid metabolism (Fig. 5C).

### 3.6. Immune-related characteristics of high and low-risk score groups

**Figure 6.**
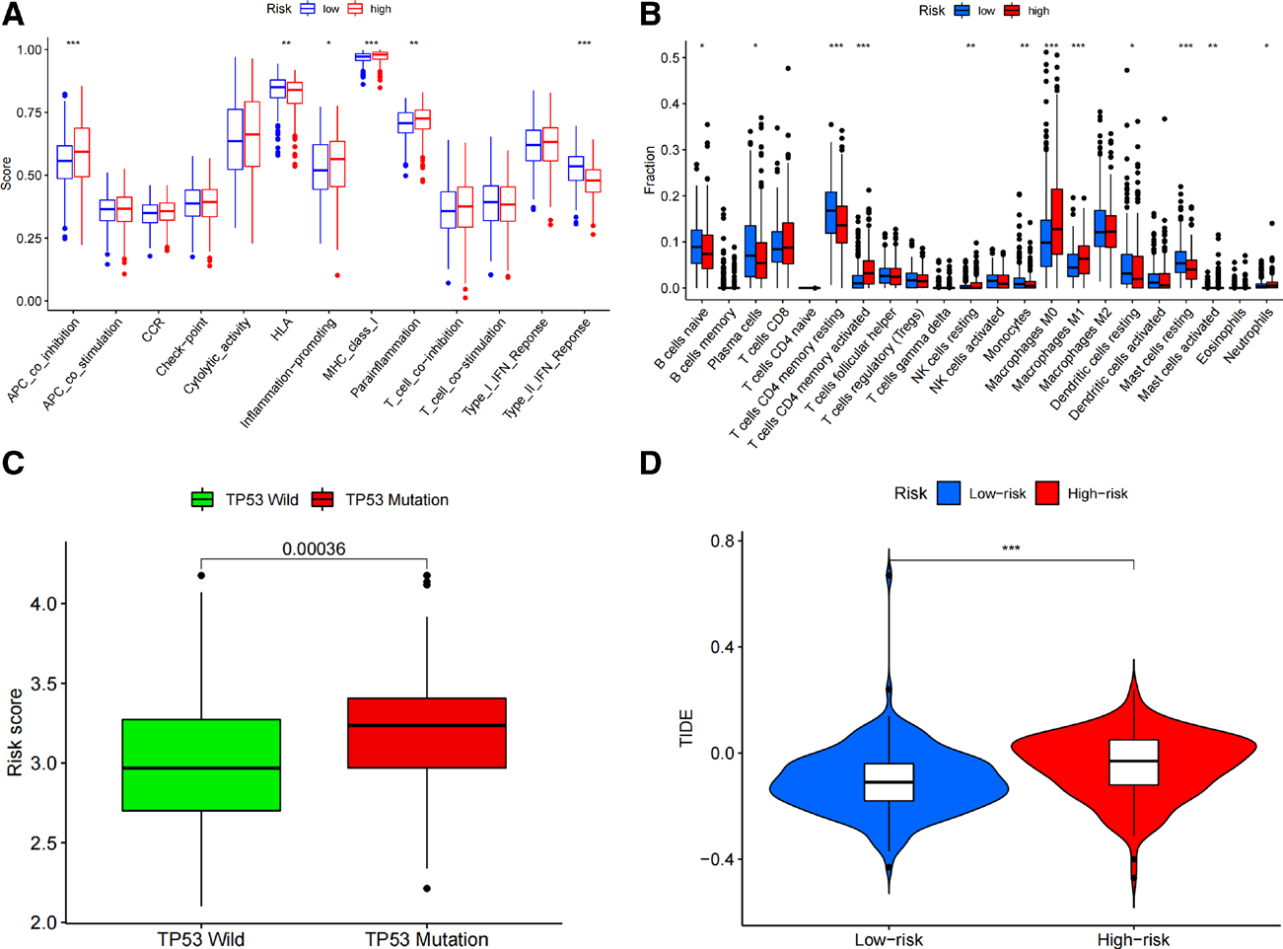
The role of fatty acid-related metabolic models in immunotherapy. (A) The differences of the abundance of immune infiltration between high-risk and low-risk score groups. (B) Immune function analysis between high-risk and low-risk score groups. (C) Relationship between TP53 and risk scores. (D) The prediction of the immune dysfunction and exclusion in high- risk and low-risk score groups.

The low-risk score group had a greater abundance of immune infiltrating cells, including B lymphocytes, CD4 + memory T lymphocytes, mast cells, and neutrophils. Type II interferon response and HLA were activated in the low-risk score group. While M0 macrophages, M1 macrophages, pro-inflammatory response, paraneoplastic, and inhibitory antigen-presenting cell responses were activated in the high-risk score group (Fig. 6A and B). TP53 was 1 of the most tumor suppressor genes associated with human tumor. The risk score of the TP53 wild was higher than that of the TP53 mutant type (*P* = .00036, Fig. 6C). The higher the risk score, the higher the TIDE and the greater the probability of immune escape (*P* < .001, Fig. 6D).

### 3.7. Protein Interaction Network Analysis of Differential Genes

**Figure 7.**
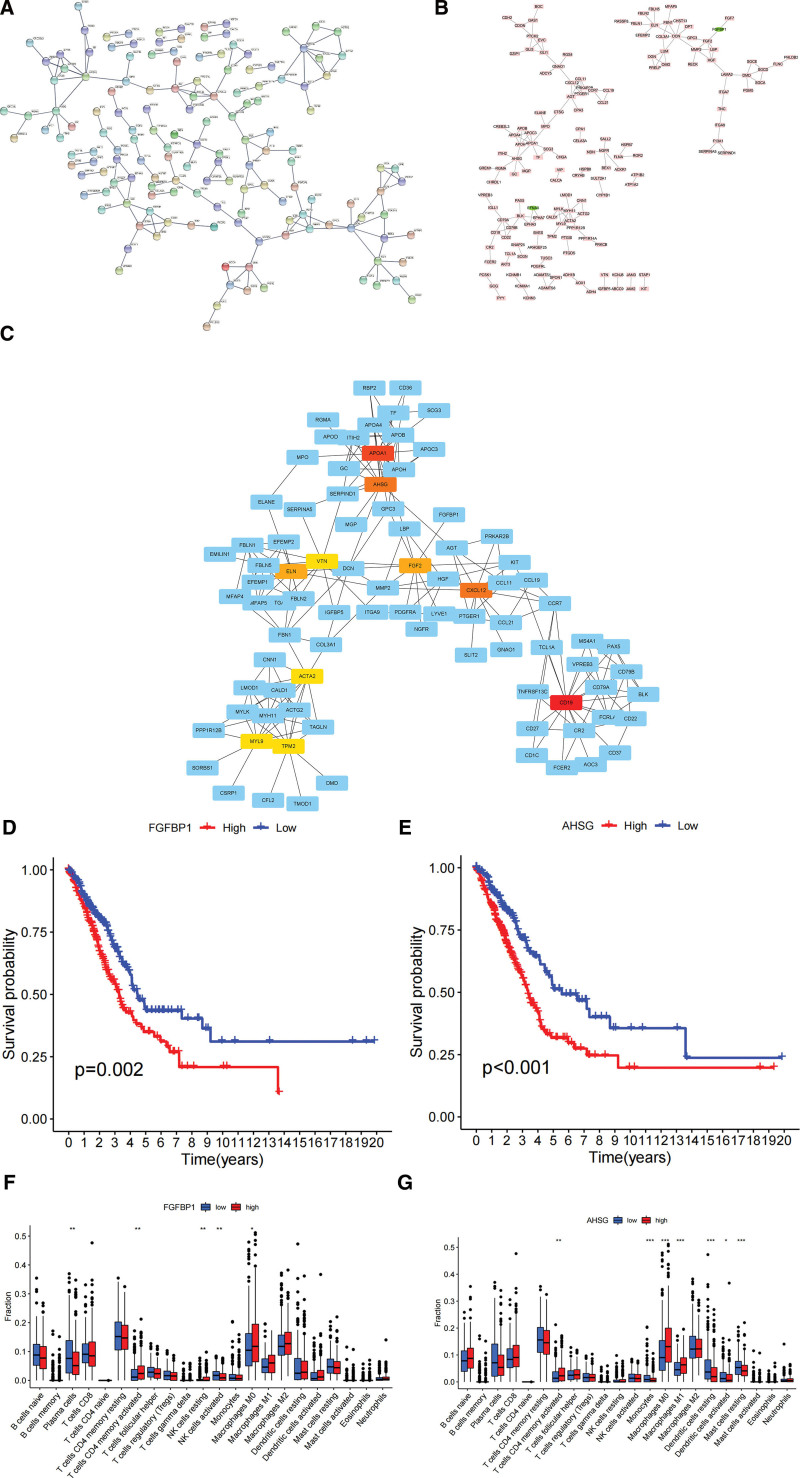
Identification and analysis of core genes. (A) Protein interactions network in String database. (B) Protein interaction network by Cytoscape. (C) Hub genes identified by cytoHubba. (D, E) Core genes with poor prognosis. (F, G) Relationship between FGFBP1, AHSG and immune infiltrating cells.

We used the String online database to analyze the protein-protein interactions (PPI) of DEGs between high-risk and low-risk groups (Fig. 7A). We used Cytoscape software to visualize the PPI data (Fig. 7B). CytoHubba was used to identify core genes of DEGs, including MYH11, ELN, DCN, FGF2, FGFBP1, CXCL12, AGT, APOA1, AHSG, APOH, APOA4 (Fig. 7C). Survival analysis revealed that MYH11, ELN, DCN, FGFBP1, CXCL12, APOA1, AHSG, and APOH were associated with patient prognosis. FGFBP1 (*P* = .002) and AHSG (*P* < .001) were associated with poor prognosis of patients (Fig. 7D and E). The distribution of immune infiltrating cells in FGFBP1 and AHSG was statistically different (*P* < .05). M0 macrophages and CD4 + memory T lymphocytes were enriched in the high FGFBP1 expression group. Plasma cells were highly expressed in the low FGFBP1 expression group (Fig. 7F). M1 macrophages and M0 macrophages were enriched in the high AHSG expression group. Dendritic cells and mast cells were enriched in the low AHSG expression group (Fig. 7G).

## 4. Discussion

It is well known that 1 of the characteristics of tumor cells, namely metabolic dysregulation, is associated with abnormal cell biological behaviors such as cell growth, angiogenesis, proliferation and invasion.^[5]^ In recent years, in addition to dysregulated glucose metabolism, abnormal fatty acid metabolism has received increasing attention as a feature of metabolic dysregulation in cancer.^[11]^ This metabolic pathway is involved in energy production, membrane synthesis and signal transduction during tumorigenesis and progression.^[11]^ molecular features of LUAD, a molecularly heterogeneous malignancy, are closely related to tumor biological behavior.^[12]^ Currently, genetic features associated with predicting prognosis have become a hot topic in cancer research. He et al constructed a multi-fatty acid metabolism gene prediction model for liver cancer using TCGA data and validated the model using the International Cancer Genome Consortiumdatabase.^[13]^ Li et al used LASSO-Cox regression analysis on TCGA data constructed a predictive model for fatty acid metabolic signature of colorectal cancer and validated it on GEO data.^[10]^ Few studies have yet focused on the relationship between lung adenocarcinoma and fatty acid metabolism genes. Therefore, we developed a predictive model for lung adenocarcinoma risk based on genes related to fatty acid metabolism, which can be used to assess the prognosis of patients.

In this study, lasso-Cox regression analysis was used to establish prognostic risk models for 11 genes associated with fatty acid metabolism in the TCGA and GEO cohort. The prognostic risk models were used to evaluate the survival of LUAD patients in the train set. There existed differences in OS between the high-risk and low-risk score groups. We obtained the same satisfactory results in the test set. Multivariate COX regression analysis and the prognostic risk model were independent prognostic factors. Functional analysis of DEGs between high-risk and low-risk score groups confirmed that oncogenic signaling and metabolic alterations were closely linked in the carcinogenesis process. Fatty acids can not only alter cell membrane components and become inflammatory mediators regulating immune cells but also further participate in the pathological process of tumors by participating in intracellular signaling regulating immune networks.^[14]^ Lipid droplet accumulation is a characteristic of chemotherapy-resistant tumor cell lines.^[15]^ A long-chain fatty acyl coenzyme A synthase (triscxinC) inhibits fatty acid activation and blocks lipid droplet production (11). Mutant TP53 can promote tumor cell proliferation, migration and invasion, enhance tumor drug resistance, destroy normal tissues, and promote tumor cell metabolism.^[16]^ We found the risk scores of the mutant TP53 group was higher than those of wild-type TP53 group, and AMP-activated protein kinase was found to be a binding site for TP53, inhibiting fatty acid synthesis by phosphorylating and inhibiting acetyl coenzyme A carboxylase (ACC) and SREBP-1.^[17]^ Recently, it has been found that TP53GOF mutants can not only promote pathological tumor progression but also increase fatty acid synthesis by inhibiting AMP-activated protein kinase. Therefore, the fatty acid metabolic pathway may serve as a therapeutic target for LUAD, especially for patients with TP53 mutations.

Immune infiltrating cells are key information to predict prognosis and immunotherapy response of various cancers. Therefore, we analyzed the abundance of immune infiltrating cells between high-risk and low-risk score groups. The low-risk score group had more immune infiltrating cells, including CD4 + memory T lymphocytes, B lymphocytes, mast cells, neutrophils, monocytes, dendritic cells, and Type II interferon response. The low-risk group was strongly associated with the stability of the tumor microenvironment. Under the TIDE algorithm, the efficacy of immunotherapy was better in the low-risk score group than in the high-risk score group. It has been found that high serum cholesterol-induced accumulation of cholesterol in natural killer cells stimulates their effector function to protect hepatocellular carcinoma cells in a mouse model.^[18]^ Altered plasticity metabolism in cancer cells results in lipid-rich TME that shapes the composition and function of immune cells and ultimately protects tumor growth from immune responses. All evidence suggests that the prognostic risk model can provide valid information for predicting LUAD immunotherapy.

Significant differences in OS between high and low-risk score groups necessitated the exploration of hub genes between the 2 subgroups. AHSG and FGFBP1 are considered essential genes and associated with poorer prognoses. AHSG is synthesized by hepatocytes and secreted into the circulatory system and is involved in various pathological processes, such as insulin resistance and regulation of bone metabolism.^[19]^ It was found that AHSG was endocytosed by tumor cells, enhancing exosome secretion, then promoting tumor cell attachment, motility, and invasion.^[20]^ AHSG is a significant driver of growth in lung cancer.^[21]^ FGFBP1, as a secreted protein, can be bound to the extracellular matrix to release fibroblast growth factor and is associated with tumor angiogenesis, cancer growth, and metastasis.^[22,23]^ In colorectal and pancreatic cancers, up-regulated FGFBP1 promotes angiogenesis and development and is associated with poor prognosis of tumors.^[24]^

The importance of clinical stage of tumors in influencing patient prognosis cannot be overstated. In the present study, fatty acid metabolism-related prognostic indicators continued to play an independent role in the prognosis of lung adenocarcinoma patients after adjusting for traditional clinical variables, suggesting the potential of this feature to improve the predictive power of traditional clinical variables. Therefore, we constructed a column line graph integrating variables such as fatty acid characteristic indicators and clinical staging.

## 5. Conclusion

In this study, We use a fatty acid-related prognostic risk model to assess fatty acid metabolic overall patterns with LUAD. The risk score correlates with patient prognosis and can be used to predict the sensitivity of chemotherapy. In addition, risk scores can effectively guide clinical practice and assess the feasibility of immunotherapy, realizing more personalized clinical practice. These provide a new idea for new therapeutic tools.

## Author contributions

**Conceptualization:** Wei Ye.

**Methodology:** Wei Ye.

**Software:** Wei Ye, Xingxing Li.

**Validation:** Wei Ye.

**Writing – original draft:** Wei Ye, Xingxing Li.

**Writing – review & editing:** Wei Ye.

## Supplementary Material


